# Toward cardiac tissue characterization using machine learning and light-scattering spectroscopy

**DOI:** 10.1117/1.JBO.26.11.116001

**Published:** 2021-11-02

**Authors:** Nathan J. Knighton, Brian K. Cottle, Sarthak Tiwari, Abhijit Mondal, Aditya K. Kaza, Frank B. Sachse, Robert W. Hitchcock

**Affiliations:** a University of Utah, Department of Biomedical Engineering, Salt Lake City, United States; b University of Utah, Nora Eccles Harrison Cardiovascular Research and Training Institute, Salt Lake City, United States; c Boston Children’s Hospital, Harvard Medical School, Department of Cardiac Surgery, Boston, United States

**Keywords:** optical biopsy, light-scattering spectroscopy, machine learning, cardiac, spectral clustering, convolutional neural network

## Abstract

**Significance:** The non-destructive characterization of cardiac tissue composition provides essential information for both planning and evaluating the effectiveness of surgical interventions such as ablative procedures. Although several methods of tissue characterization, such as optical coherence tomography and fiber-optic confocal microscopy, show promise, many barriers exist that reduce effectiveness or prevent adoption, such as time delays in analysis, prohibitive costs, and limited scope of application. Developing a rapid, low-cost non-destructive means of characterizing cardiac tissue could improve planning, implementation, and evaluation of cardiac surgical procedures.

**Aim:** To determine whether a new light-scattering spectroscopy (LSS) system that analyzes spectra via neural networks is capable of predicting the nuclear densities (NDs) of ventricular tissues.

**Approach:** We developed an LSS system with a fiber-optics probe and applied it for measurements on cardiac tissues from an ovine model. We quantified the ND in the cardiac tissues using fluorescent labeling, confocal microscopy, and image processing. Spectra acquired from the same cardiac tissues were analyzed with spectral clustering and convolutional neural networks (CNNs) to assess the feasibility of characterizing the ND of tissue via LSS.

**Results:** Spectral clustering revealed distinct groups of spectra correlated to ranges of ND. CNNs classified three groups of spectra with low, medium, or high ND with an accuracy of 95.00±11.77% (mean and standard deviation). Our analyses revealed the sensitivity of the classification accuracy to wavelength range and subsampling of spectra.

**Conclusions:** LSS and machine learning are capable of assessing ND in cardiac tissues. We suggest that the approach is useful for the diagnosis of cardiac diseases associated with changes of ND, such as hypertrophy and fibrosis.

## 1 Introduction

Optical technologies for medical diagnosis have advanced rapidly over the last several decades. A broad category of these new technologies characterized as “optical biopsies” uses the interaction of light with tissue for its *in-vivo* characterization and disease diagnosis—in effect replacing traditional biopsy based on tissue extraction. These light-tissue interactions are leveraged for tissue characterization by various optical modalities, including fluorescence imaging, multi-photon microscopy, spectroscopy, and tomography. Due to their diagnostic effectiveness combined with miniaturization and low cost, these technologies show promise as an effective means to improve patient care while reducing expense.

Miniaturization of optical components such as lenses and the development of coherent optical fiber-optic bundles have enabled the integration of optical technologies into catheters for use in a variety of previously unattainable regions, such as *in-vivo* pulmonary and cardiac applications. Fiber-optic confocal microscopy (FCM) and optical coherence tomography (OCT) are examples of optical technologies being explored for use in the heart.^1^^–^^4^ These imaging modalities show promise in interventional cardiology and cardiac electrophysiology.^5^ OCT is also being explored for the guidance of interventional cardiac procedures such as stent placement.^2^^,^^6^^,^^7^ Clinical studies and related research with FCM have shown the ability to identify conductive tissue regions during congenital heart surgery, e.g., the sinoatrial and atrioventricular nodes.^8^^,^^9^ Although these technologies have been shown to provide useful information during cardiac procedures, the significant cost and technical complexity associated with their implementation hinder widespread adoption. Additionally, shallow imaging field depths limit the application of these technologies to only a few specific use cases in the heart.^10^^,^^11^

Spectroscopic approaches using light in the visible and near-infrared spectrums are promising alternatives that could decrease overall cost and complexity while increasing the depth of tissue characterization by leveraging the physics of light transport and scattering in tissue.^1^^,^^10^^,^^12^ Although spectroscopic approaches are capable of gathering information from deeper within tissue samples, they result in significantly lower spatial resolution when compared to FCM and OCT. Of the numerous spectroscopic approaches that have been developed, this study is focused on implementing light-scattering spectroscopy (LSS), which is an established approach used in research laboratories and clinical settings to study a variety of systems throughout the body.^13^^–^^15^ LSS measures the scattering of light of different wavelengths in a medium with discrete particles. Several properties of the particles within a medium, such as density, size, and shape, contribute to the scattering behavior. Gathering spectra from tissue samples can yield information about the size and distribution of nuclei as well as other characteristics of the tissue, such as chromatin content.^14^

Many cardiac diseases such as myocarditis, amyloidosis, and other cardiomyopathies as well as allograft rejection are diagnosed by observing and quantifying microstructural abnormalities. The current gold standard for diagnosis of these diseases is retrieving a biopsy of cardiac tissue, a procedure associated with significant risk. LSS as a means of characterizing abnormal microstructural changes in cardiac tissue has the potential to decrease the risk and harm associated with diagnosing and monitoring these diseases by gathering information from the tissues in a non-destructive manner. An important foundation that needs to be established for the development of LSS toward application in these cardiac diseases is the sensitivity of the LSS spectra to changes in the scattering profile of tissue and its correlation with changes in the underlying tissue, such as the density of myocyte nuclei.

We investigated the ability of LSS to identify the nuclear densities (NDs) of the myocardium in combination with machine learning. Machine learning is increasingly utilized to overcome challenges related to analyzing complex signals such as spectra.^16^^–^^18^ Previously, our research group demonstrated the ability of a machine learning approach, i.e., a convolutional neural network (CNN), to detect fibrosis in FCM images taken in a beating heart *in situ*.^19^^,^^20^ Other previous studies explored machine learning for cardiac ablation lesion quantification^21^ and diagnosis of ischemic heart disease.^17^^,^^22^

Here, we introduced a novel system for broad-spectrum LSS for applications in cardiology and cardiac surgery. This system measured spectra from cardiac tissues of an ovine model spanning a large range of gestational ages, including pre-term and adult. Previous work on cardiac tissues from ovine models showed a decrease in ND with age.^23^^,^^24^ Here, we applied this finding to evaluate the ability of machine learning to identify the relative ND in these ovine hearts. Spectra were analyzed using unsupervised and supervised machine learning methods, i.e., spectral clustering and 1D CNNs. We used fluorescent labeling confocal microscopy and image processing to measure NDs in cardiac tissues. These values were then used as ground truth for training neural networks to predict ND from measured spectra.

## 2 Methods

### 2.1 LSS System

**Fig. 1:**
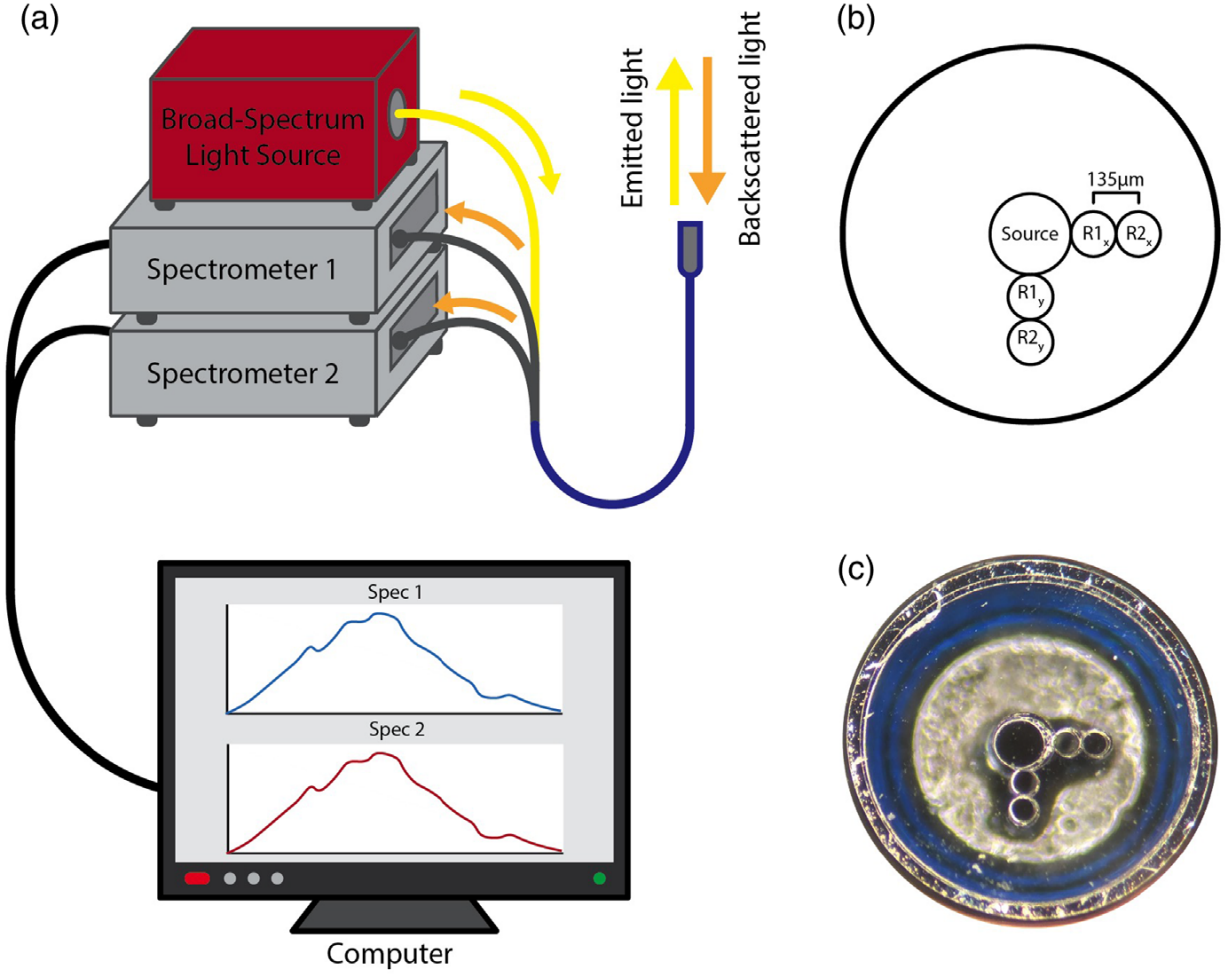
Spectroscopy system. (a) System overview. Broad-spectrum light was applied to tissue using an illumination fiber. Backscattered light was collected by collection fibers and measured by Czerny-Turner type CCD spectrometers. (b) Schematic view of fiber arrangement showing central illumination fiber and orthogonal fiber pairs for collecting light. (c) Tip of prototype LSS probe.

A customized LSS setup was developed for application in cardiac tissue. A custom-built spectroscopy probe (Berkshire Photonics, Washington Depot, CT) was used to both illuminate the tissue with broad-spectrum light and gather light from the tissue. The setup shown in Fig. 1 includes a stabilized tungsten-halogen broad-spectrum light source (SLS201L/M, Thorlabs, Newton, New Jersey) providing light in the 350- to 2000-nm range, two Czerny-Turner type charge-coupled diode (CCD) spectrometers (CCS175/M, Thorlabs), a computer with spectrum acquisition software (OSA, Thorlabs), and the spectroscopy probe.

The probe utilized a single 200-μm core diameter central illumination fiber (FVP200220240, Molex, Phoenix, Arizona) accompanied by four 100-μm core diameter light collection fibers (FVP100110125, Molex). These four light collection fibers were fixed into positions along two orthogonal radial arms adjacent to the central illumination fiber, with two of the four fibers positioned along each of the arms. Figures 1(b) and 1(c) show the arrangement of the fibers at the tip of the probe. The two fibers along each arm were positioned with center-to-center distances of 210 and 345  μm from the illumination fiber. The fibers positioned 210  μm from the central illumination fiber were connected to a single CCD spectrometer, and the fibers positioned at 345  μm were connected to a separate spectrometer. These two sets of fibers are referred to collectively as the R1 and R2 fibers, respectively. The arrangement of two sets of fibers equidistant yet orthogonal in relation to the central illumination fiber was chosen to mitigate the effects of optical anisotropy inherent in cardiac tissue.^25^ The tip of the probe comprises a 1.7-mm outer diameter stainless steel tube and a 1-mm thick laser-cut quartz glass end cap into which the illumination and absorption fibers are fixed using epoxy. A prior study established the capabilities of this system regarding quantifying depth, arrangement, and composition of fibrotic cardiac tissues.^20^

### 2.2 Spectroscopy of Cardiac Tissue

**Fig. 2:**
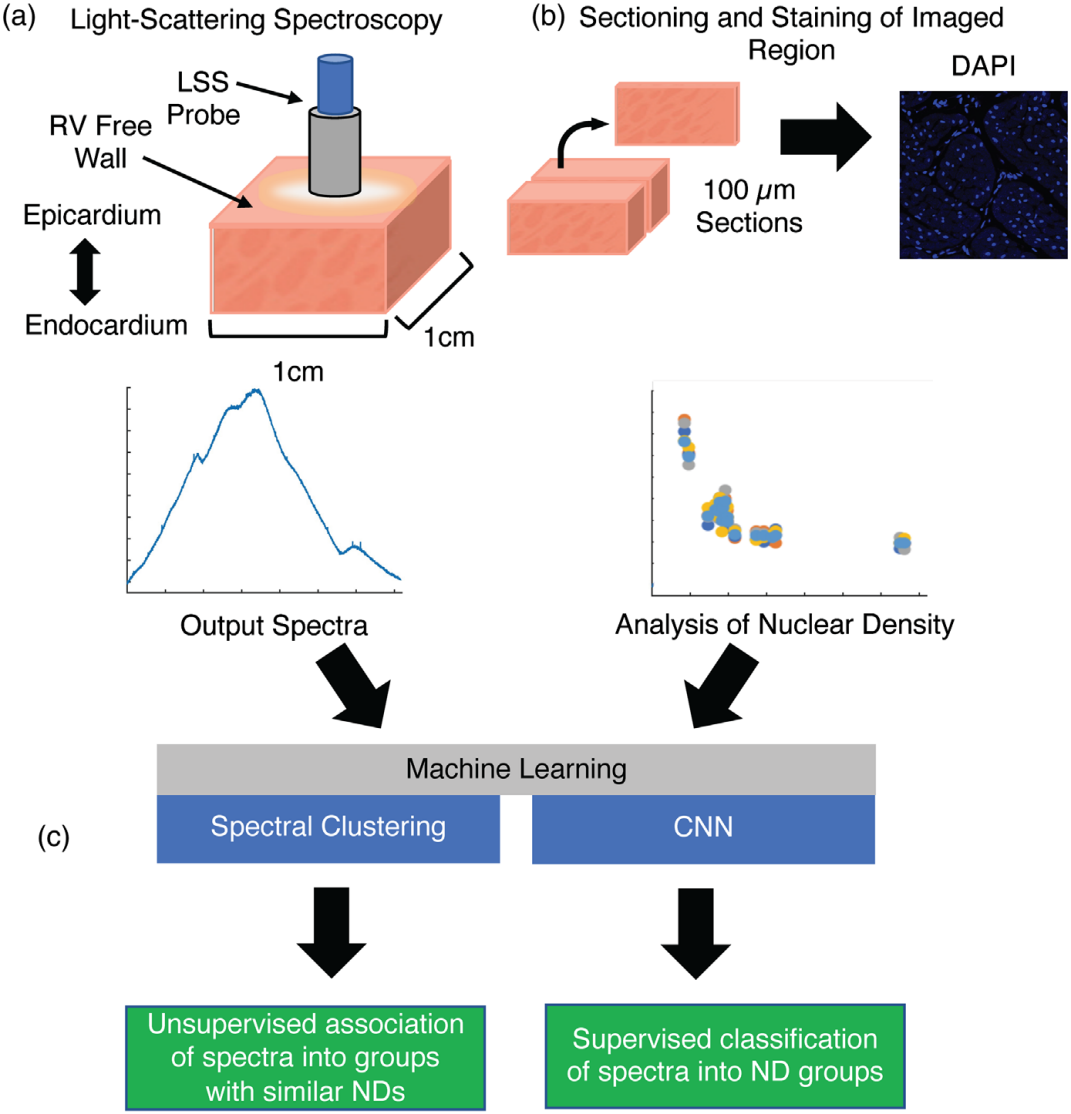
Experimental design. (a) Spectra were gathered from $∼1\;{\mathrm{cm}}^{3}$ transmural samples of fixed ovine hearts. (b) A 100-μm thick section from the transverse center of the tissue sample at the same location as for spectroscopy. The section of tissue was stained with WGA and DAPI. The spectra from (a) and measured ND from (b) were applied for (c) machine learning. Also, spectra and measured ND were used to test the prediction of NDs.

The LSS system was used to gather spectra from ovine ventricular tissue according to the process outlined in Fig. 2. All animal usage was approved by the Institutional Animal Care and Use Committee (IACUC) at Boston Children’s Hospital. We obtained an $∼1\;{\mathrm{cm}}^{3}$ transmural tissue sample from the right ventricular free wall (RV) of formalin-fixed hearts from 18 animals with gestational ages ranging 4.3 to 56 months. Two additional samples from the left ventricular free wall and ventricular septum of the two youngest hearts brought the total sample size to 22. Samples were placed on a black foam pad and kept submerged in phosphate-buffered saline (PBS) before and during spectroscopy. Black open-cell foam prevented backscattering of photons after passing through the sample. Samples were examined using LSS to identify changes in ND. The LSS probe was positioned on the epicardial surface at multiple locations to gather 20 spectra for each sample. The spectra were gathered with an integration time of 200 ms. Spectra were recorded with full width at half maximum resolution of 0.6 nm in the wavelength range of 500 to 1100 nm. Following acquisition, the spectra were saved for analysis. Tissue samples were stored in PBS for histology. Each spectrum was normalized to its mean intensity. The spectra were then filtered using a 1D gaussian filter with a standard deviation of 20 measures (∼3.3  nm) and a kernel size of 80 measures.

### 2.3 Histology

After spectral measurements, tissue samples were sectioned within the probed region at the center of each sample. A vibratome (Leica Model VT1200S, Wetzlar, Germany) was used to section at 100-μm thickness perpendicular to the epicardial surface and parallel to the transmural plane of the ventricular wall. This orientation was chosen to include light scattering structures throughout the thickness of the ventricular wall. After sectioning, glycoconjugates of the extracellular matrix and glycoproteins of cell membranes were labeled using wheat germ agglutinin (WGA) conjugated to a fluorophore (WGA CF488A Conjugate, Biotium, Fremont, California). Cell nuclei were stained with 1  μM 6-diamidino-2-phenylindole (DAPI, D3571, Life Technologies, Carlsbad, California) according to a previously established protocol.^26^ All sections were washed in PBS and mounted on a glass slide with Fluoromount-G (#17984-25, Electron Microscopy Science, Hatfield, Pennsylvania).

A Leica SP8 confocal microscope with a 40× oil immersion lens (numerical aperture 1.3) was used to scan each section. Two-dimensional tile scans with a pixel size of 0.2  μm spanning 695  μm×2467  μm were performed for the capture of cardiac tissue, including the epicardium. DAPI and the WGA CF488A Conjugate were excited with a 405 nm and 488 laser, respectively.

Two or more different tile scans from each sample were used to calculate the ND. Manual segmentation was performed using the Fiji/ImageJ image processing software package.^27^ Histogram-based thresholding (mode + 2 standard deviations) isolated nuclei in the DAPI images. Gaussian blurring with a sigma of 2 followed by erosion and dilation removed small segments. Connected nuclei were separated using a watershed function. Visual inspection of segmented images validated segmentation methodology. Automated segmentation and counting were performed to validate the ND values gathered from the manual segmentation. This was performed using Python 3.8.2 while following the same operands as the manual segmentation. The image processing was primarily done using the package scikit-image (0.17.2).^28^ For both the manual and automated methods, ND was calculated by dividing the number of detected nuclei by the tissue area. The average ND produced by the manual segmentation for each tissue sample provided the ground truth for machine learning.

### 2.4 Assessment of the Spectra–ND Relationship by Cluster Analysis

Unsupervised and supervised machine learning approaches were used to explore whether spectra gathered from the LSS system allowed for differentiation between the ND of the preparations. We employed MATLAB (2019b, MathWorks, Natick, Massachusetts) and its Deep Learning Toolbox for unsupervised learning evaluation of the correlation between LSS spectra and the measured ND.

We explored normalized, filtered, and concatenated spectra from the paired collection fibers. Each spectrum was annotated with the ND of the sample from which it was measured. We applied the MATLAB function *spectralcluster* to identify clusters of spectra from preparations of different NDs using the Euclidean distance metric. From this spectral clustering analysis, we generated a similarity graph between nodes of the first and second principal components of the spectra.^29^ Analysis of the eigenvalues from the spectral cluster function indicated the spectra fell into five distinct groups. We utilized the MATLAB function *gscatter* to visualize the spread of the spectral set, with the resulting groups identified by color. Groups were associated with the NDs of the spectra contained in each group. The principal components analysis (PCA) was performed, and the first two principal components were used to visualize any potential groups within the dataset. A one-way ANOVA and Tukey-Kramer post hoc test using a significance level of 0.05 was used to identify differences between the clustered spectra. A box and whisker plot was created from these data, which compared the NDs associated with the spectrum within each group produced from the spectral clustering. Significant differences between clusters were indicated by brackets.

### 2.5 Assessment of the Spectra–ND Relationship by CNN

We applied CNNs to differentiate NDs based on spectra from LSS. This work was built upon our previous work on identifying fibrosis from in vivo confocal imaging.^19^ Our software framework was based on Python scripts and the Keras neural network API (2.3.1)^30^ with Tensorflow (2.2.0).^31^ We designed a CNN to classify the spectra into three groups of ND 0 to 2000, 2000 to 3800, and above $3800\text{ nuclei}/{\mathrm{mm}}^{2}$.

The CNNs were systematically trained and validated using a form of k-fold cross-validation called leave-one-out cross-validation (LOOCV).^32^ This approach entailed creating 22 different unique datasets, each of which removed the spectra gathered from one of the 22 different samples. This removed sample of spectra was separated for use as a testing dataset for each fold. Another sample was also randomly selected from the then remaining 21 samples in each fold to be removed and used as a validation dataset to guide hyperparameter optimization. In all, for each LOOCV performed, 22 networks were trained, each on a training dataset that consisted of the spectra from 20 different samples, less the validation and training samples. To address and reduce the likelihood of the networks overfitting the datasets, initial network hyperparameters were selected that minimized the number of trainable parameters.

Two tests were performed to determine the potential sensitivity of the networks to specific wavelength ranges and to also determine the sensitivity of the networks to the resolution of the spectra. An initial network configuration was selected (Table 1). Hyperparameter variations were later performed using this network structure as a base. To determine the sensitivity of the networks to wavelength ranges, three training sessions of LOOCV were performed on spectra consisting of wavelengths 500 to 700 nm, 700 to 900 nm, and 900 to 1100 nm, respectively. One-way ANOVA and posthoc test using Fisher’s least significant difference method with a level of 0.05 were applied to determine any significant differences between the accuracies reported for each wavelength range. To determine the sensitivity of the networks to the resolution of the data, 15 LOOCV training sessions were performed using spectra with resolutions reduced by 15 different scales, K, distributed logarithmically between 10 and 1000. The spectra were downsampled using a 1D linear interpolation between every Kth point in the original spectra. When using the network structure outlined in Table 1 in conjunction with the downsampled spectra, the number of trainable parameters within the networks varied between 1755 and 135, for scales of 10 and 1000, respectively. Beyond the hyperparameters described in Table 1, both the wavelength-dependency and the resolution-reduction LOOCV training sessions were performed using a learning rate of 0.005 for all networks. Training for these sessions was terminated early if the loss did not decrease by more than 0.001, a value referred to as the “minimum delta” during parameter variations, after 100 epochs; otherwise, the training was permitted to run up to 1000 epochs. The categorical cross-entropy was calculated from the logits produced from the final layer of the network for use during the training process and post-analysis. This loss function was selected because each spectrum belongs to only one of the three ND ranges.

**Table 1:** CNN Configuration.

Layer #	Layer type	Parameters
1,2,4	1D convolutions	Filter Number: 3, Kernel Size: 5, Stride: 2, Activation: ReLU
3,5	1D Max Pooling	Stride: 2, Pool Size: 2, Padding: None
6	Softmax	Exponential activation layer for multi-class classification

Although hyperparameter variation was not performed during these training sessions, a validation sample was still removed from the training dataset in addition to the testing sample. Network weights were saved during the training process when the loss value decreased. The network weights resulting in the lowest loss value were restored at the end of the training session. The predicted classes from the testing datasets for each fold were then compared to the assigned classes for each spectrum based on ND values. From this comparison of aggregated predictions, we calculated the accuracy for the CNN topology on the entire LOOCV as follows:

$$\text{Accuracy}=\;\frac{n_{\text{correct}}}{n_{\text{total}}},$$ (1)

where $n_{\text{correct}}$ is the number of correct predictions, and $n_{\text{total}}$ is the total number of predictions.

To improve the accuracy of the predictions resulting from the LOOCV, the above training process was altered to introduce hyperparameter variations within each training fold. Multiple networks were trained using the base topology outlined in Table 1 to maintain a low number of trainable parameters while varying the learning rate. The resolution of the spectra was also reduced by a factor of 50 using the methods described previously. This reduction decreased the number of trainable parameters while still maintaining most of the variations within the spectra that remained after filtering and produced a network topology with 441 trainable parameters. Ten different learning rates, logarithmically distributed between 0.0001 and 0.01, were used as well as four different minimum delta values: 0.001, 0.003, 0.005, and 0.007. The randomly selected validation sample associated with each network was then used to determine the validation accuracy and loss values of the network. The optimal learning rate and minimum delta for each network were selected based on the highest accuracy value reported. In cases where more than one hyperparameter set resulted in the same highest accuracy value, the hyperparameter set with the lowest validation loss score between them was selected. Once optimal hyperparameter values were selected for each of the 22 folds of the LOOCV, the optimal networks for each fold were used to predict the classes of their respective testing datasets. The average accuracy value of these predictions was then calculated using Eq. (1).

## 3 Results

### 3.1 Spectroscopy of Tissue Samples

**Fig. 3:**
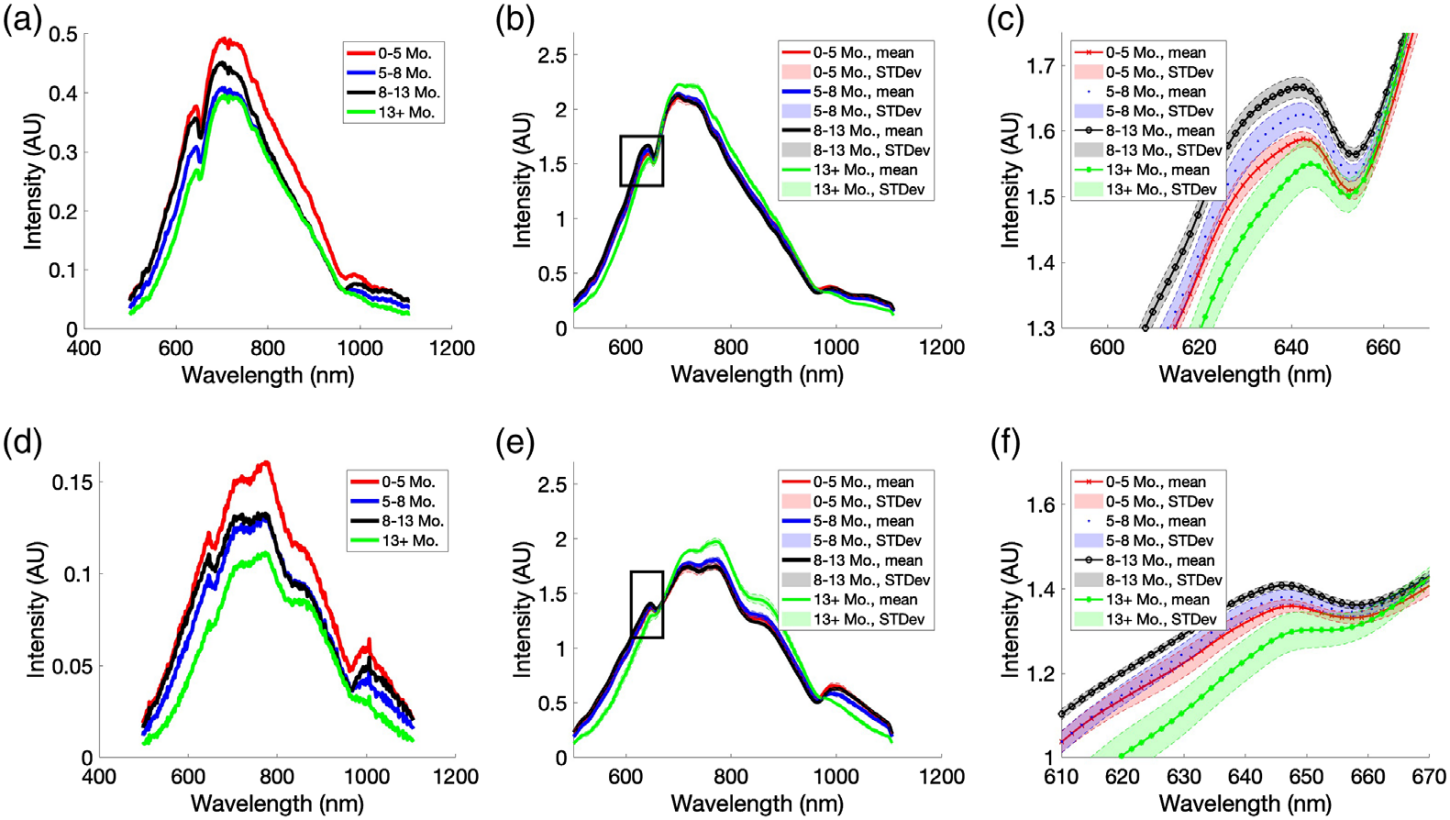
Comparison of spectra for selected gestational ages within the ranges of 0-5, 5-8, 8-13, and 13+ months. (a) Raw spectra from R1. (b) Averaged spectra for each age range from R1 after normalization and filtering. The black box indicates the region highlighted in 3C. (c) Enlarged view of the region indicated with a black box in 3B. This region was selected as it shows where the four age ranges differ significantly from each other. (d) Raw spectra from R2. (e) Averaged spectra for each age range from R2 after normalization and filtering. The black box indicates the region highlighted in 3F. (f) Enlarged view of the region indicated with a black box in 3E. This region was selected as it shows that spectra from tissues of different age ranges differ from each other.

We gathered 440 spectra from two collection fibers from 22 samples. Raw spectra displayed a moderate degree of variability in intensity within samples and in-between samples. Spectra from R1 and R2 were different with respect to intensity range and shape [Figs. 3(a) and 3(d)]. After normalization to their respective means, spectra from an individual sample exhibited a high correlation with the other spectra from the same sample ($R^{2}\geq 0.999$). Differences between spectra from different samples were subtle ($R^{2}=0.996\pm 0.002$). Figures 3(b) and 3(e) show mean spectra with standard deviations from four representative age groups. Figures 3(c) and 3(f) highlight regions within the spectra that present differences across the dataset associated with age.

### 3.2 Histology and Determination of ND

**Fig. 4:**
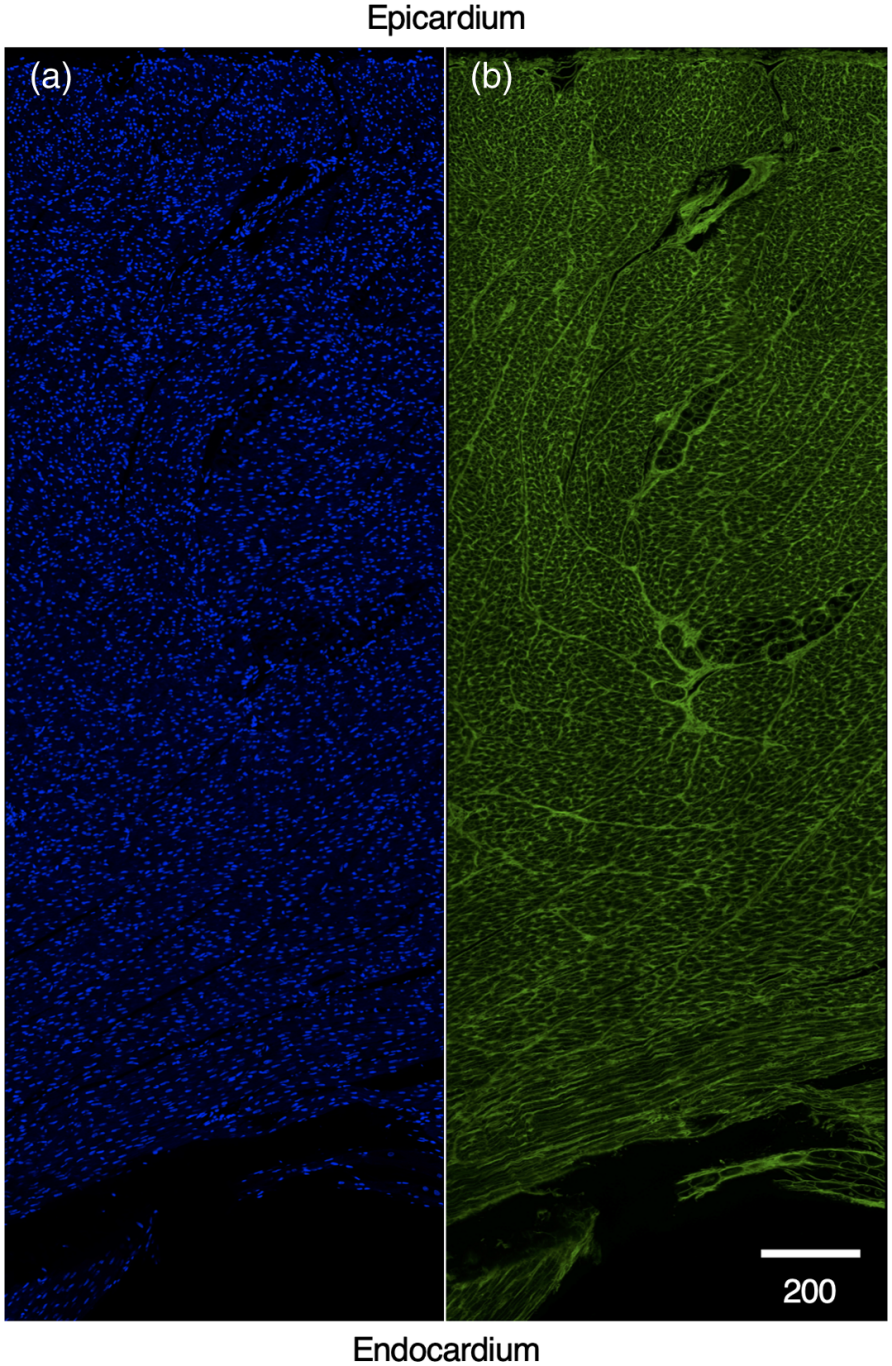
Transmural section of heart wall from a 4.3-month old animal from confocal microscopy. (a) Nuclei stained with DAPI. (b) Extracellular matrix staining with WGA.

**Fig. 5:**
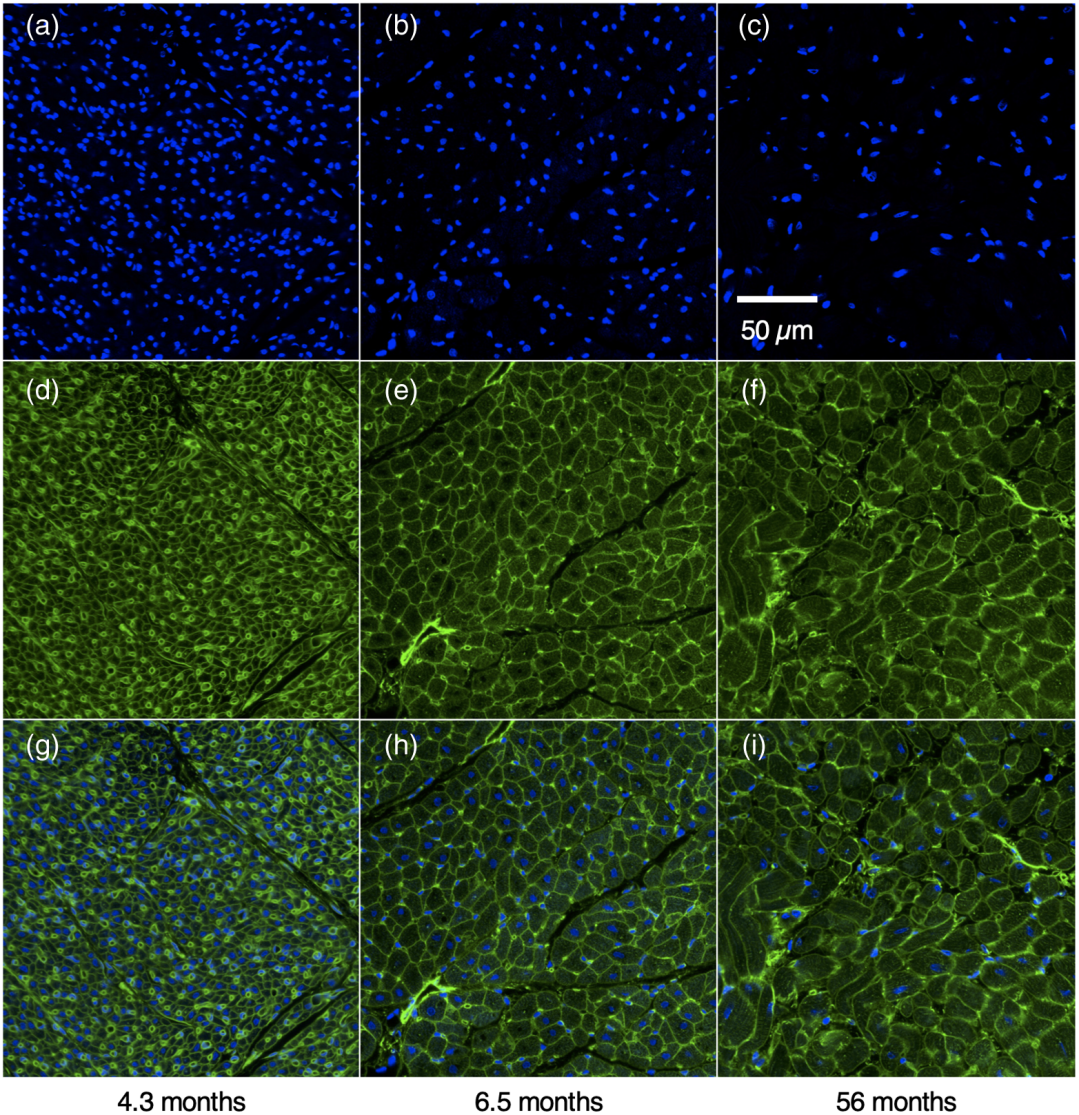
Confocal images of cardiac tissue at gestational ages of (a) 4.3, (b) 6.5, and (c) 56 months. (a)–(c) DAPI labeled cell nuclei. (d)–(f) WGA labeled extracellular matrix and capillaries. (g)–(i) Composite DAPI and WGA images.

Representative transmural confocal microscopy tile scans of RV free wall myocardium are shown in Fig. 4. Images of DAPI revealed higher ND and smaller myocytes in samples for younger animals vs. samples from older animals (Fig. 5). Decreases of ND in samples of 4.3, 6.5, and 56 months gestational age are visible in 250  μm×250  μm confocal images shown in Figs. 5(a)–5(c). Increased size of cardiac myocytes is visible from WGA images of the same samples is evident in Figs. 5(d)–5(f). Composite images are presented in Figs. 5(g)–5(i).

**Fig. 6:**
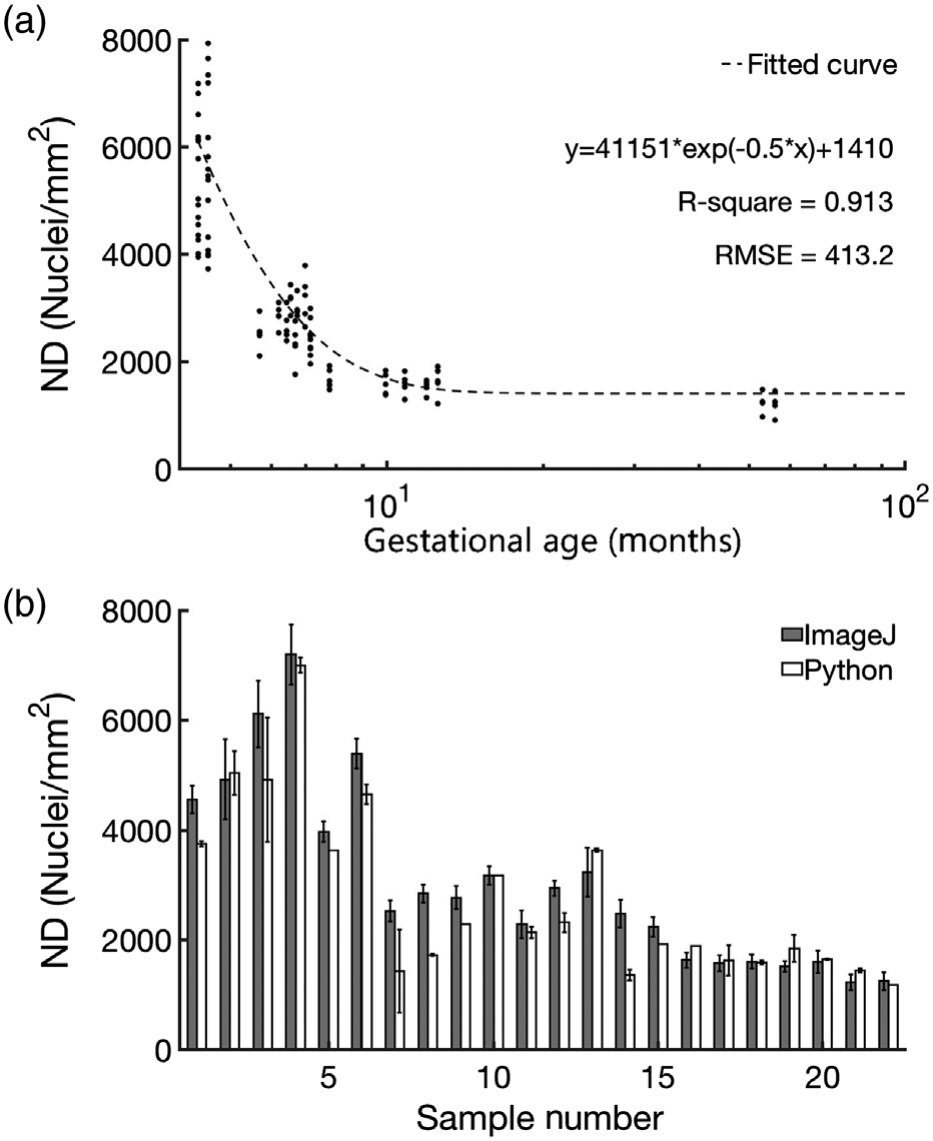
Counting of cell nuclei. (a) Scatterplot and regression fit of measured NDs relative to age. (b) Comparison of NDs from different segmentation methods.

Manual segmentation using ImageJ indicated a 72.5% difference in ND between samples of 4.3 months and 10 months gestational age and a 78.2% difference in ND from 4.3 to 56 months. Automated segmentation yielded a 65.4% and 77.8% decrease in ND for the same age differences. The reduction of ND with age in our samples is summarized in Fig. 6(a) and consistent with prior studies.^23^ ND differences between segmentation methods varied most for samples from the youngest hearts. Figure 6(b) shows the measured values for each segmentation method. The correlation between segmentation methods was high ($R^{2}=0.94$). Although the results from both the manual and automated segmentation techniques were similar, the values resulting from the manual segmentation were selected as ground truth for the duration of the study as they more closely reflected the reduction in ND over time in the literature.^22^

### 3.3 Cluster Analysis of Spectra

**Fig. 7:**
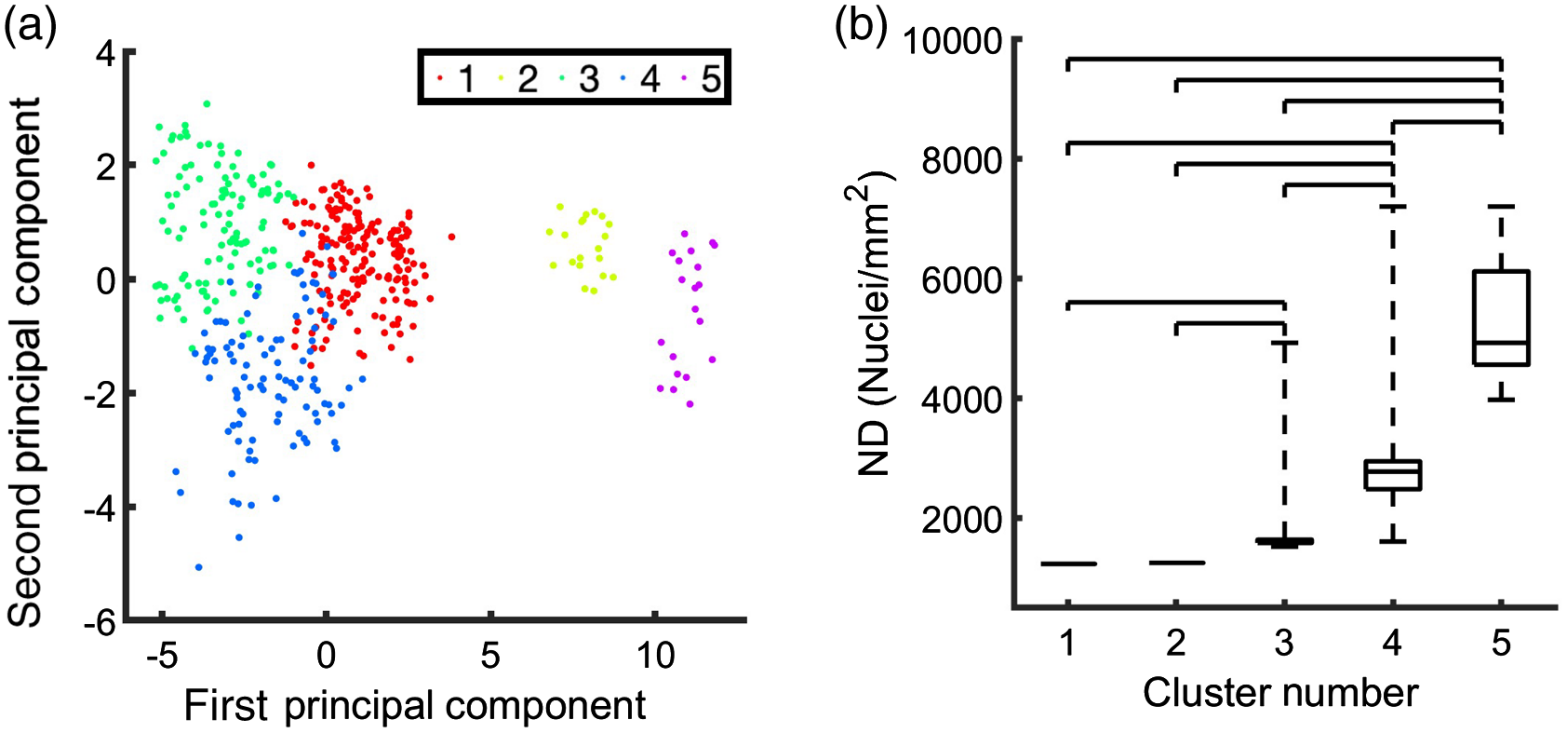
Spectral clustering of spectra measured from cardiac tissues. (a) The clustering identified 5 clusters shown in a principal component plot. (b) NDs of several identified clusters were different. Bracket identified clusters that are statistically different from one another at a 5% confidence level.

Dimensionality reduction via PCA revealed that the first two principal components accounted for ∼95% of the variance between spectra. The cluster analysis revealed five clusters [Fig. 7(a)]. Two of the clusters contained only 20 spectra, and each contained spectra from only a single sample, whereas the other three clusters contained over 100 spectra with at least five samples in each cluster. Only a small percentage of spectra were not assigned to the same cluster as the majority of the spectra from the same tissue sample. In sum, a total of 23 of the 440 spectra (∼5%) were miscategorized. Clusters were labeled 1 to 5 with increasing ND. Differences in ND were significant for many of the clusters [Fig. 7(B)]. For instance, NDs of clusters 3, 4, and 5 were different from clusters 1 and 2. Differences of ND for clusters 1 and 2, corresponding to the samples of lowest ND, were not significant.

### 3.4 CNN-Based Classification of ND Grouping

**Fig. 8:**
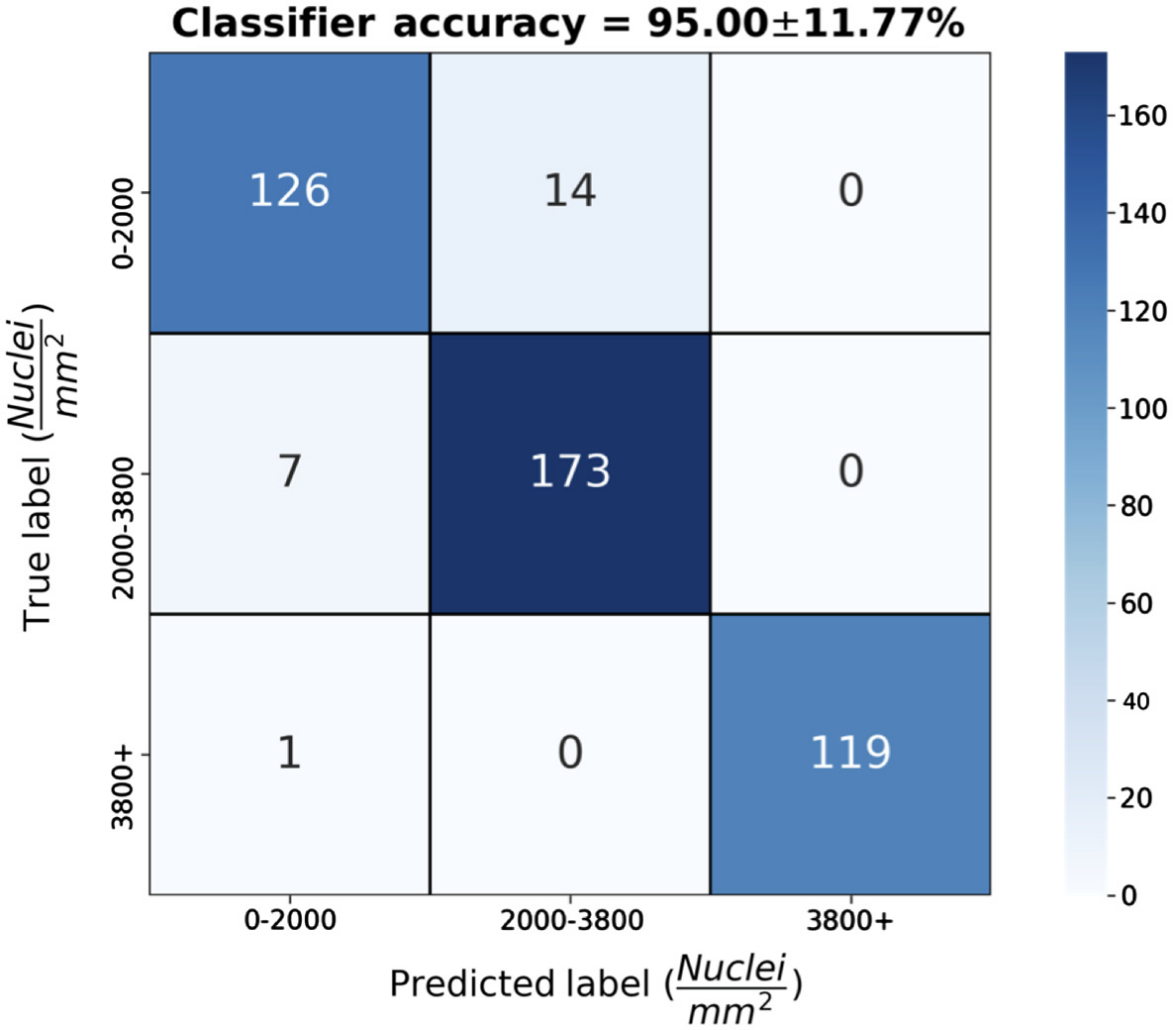
Confusion matrix of aggregated CNN predictions of NDs from spectra. The resulting classifier accuracy was higher than 90%.

We explored the sensitivity of the CNN to different wavelength ranges. Accuracies of 59±46%, 40±48%, and 76±33% resulted from the wavelength ranges 500 to 700 nm, 700 to 900 nm, and 900-1100 nm, respectively. Differences between the 500 and 700 nm and 700 and 900 nm ranges were not significant. Also, differences between the 500 and 700 nm and 900 and 1100 nm ranges also were not significant. Accuracies for the 900 and 1100 nm were higher than for 700 to 900 nm (p-value: 0.0075). The exploration of the sensitivity of the network to the resolution of the spectra resulted in accuracies ranging from 67% to 95%. There were no statistically significant differences between the LOOCV sets based on the reduction in data resolution. Two reduced resolution datasets resulted in accuracies above 90%, i.e., the datasets reduced by factors of 37 and 268. The dataset reduced by a factor of 1000 resulted in an accuracy of 83±31%. The accuracy from the optimal hyperparameters for each fold of the training process was 95±12%. Classifications of spectra for this final network are summarized in the confusion matrix in Fig. 8. Among the 22 optimized networks, all the minimum delta values of 0.007, 0.005, 0.003, and 0.001 were selected 2, 6, 7, and 7 times each. Of the possible learning rates only five, 0.01, 0.00615, 0.00378, 0.00233, and 0.00143, were selected. These learning rates occurred 5, 9, 5, 2, and 1 time, respectively.

## 4 Discussion

The presented studies suggest that the developed LSS system can predict NDs in cardiac tissue samples within predefined ND ranges based on CNNs. The studies were based on microscopy and image processing, which showed decreases in ND on the order of 70% between hearts of 4.3 to at least ten months gestational age. This decrease is consistent with previously observed values.^23^^,^^24^^,^^33^ NDs were identified by two separate machine learning approaches. Spectral clustering in a dataset of spectra identified five clusters with similar NDs. Also, a CNN allowed us to classify spectra into three predefined ND ranges with a high (>90%) accuracy.

LSS is particularly well suited to identify the change in the relative distribution of major cellular components such as nuclei, which have long been recognized as major sources of light scattering.^34^^,^^35^ The distribution of nuclei and extracellular matrix components is a hallmark of certain tissue structures and pathologies of the cardiovascular system. Changes in these distributions and structures can be expected to alter the behavior of light scattered by the tissue.

LSS relies on the wavelength-specific absorption and scattering of photons by cellular and subcellular structures as well as constituents of the extracellular matrix.^12^^,^^34^^,^^36^ The optical properties of the tissue constituents determine the type and probability of light-tissue interactions. A fraction of the photons is backscattered and detected by a spectrometer. Predominant sources of scattering are cellular membranes, mitochondria, and nuclei.

It is well understood from animal models that the ND of heart muscle decreases in maturing neonates as the cardiac myocytes grow.^23^^,^^24^ However, the total number of myocytes and the number of their associated nuclei change little. This results in the age-related change in ND that was utilized for our study assessing our LSS system.

Analysis of LSS spectra is often a time-consuming endeavor based on simplified models and mathematical simulations of light-tissue interactions.^13^^,^^15^^,^^35^ Early work on LSS systems applied to biological tissue relied on the derivation of simplified models or complex Monte Carlo simulations to identify changes in spectra that correlated to changes in the relative distribution of specific scatterers.^14^^,^^35^ Currently, a general framework does not exist for analyzing LSS spectra from cardiac tissues. Here, we used machine learning to generalize analyses of spectra. We utilized two approaches to analyze NDs based on LSS spectra, i.e., spectral clustering and a CNN. With spectral clustering, spectra were grouped independently of any human input, which revealed a relationship between spectra and ND. The advantage of this approach is the potential for automated and rapid identification of ND without the need for additional human input or preprocessing of the spectral signal. We suggest that due to the minimal preprocessing involved, our analysis approach can be extended to an LSS system capable of real-time mapping of cardiac regions based on their spectral signatures.

Our second approach for the prediction of NDs from spectra was based on CNNs and histological quantification of NDs. The high accuracy of our predictions using LOOCV demonstrated that the CNN can reliably classify spectra into predefined ND groups. CNNs perform feature extraction and dimensionality reduction intrinsically, thus reducing or removing the need for preprocessing frequently required when working with spectral data. For these reasons, CNNs are well suited to handle raw signals.^37^ This means that analysis of spectra by CNNs has the potential to replace many of the current computationally expensive approaches for preprocessing and analysis of spectra.^15^^,^^16^^,^^36^ However, we note difficulties in applying a CNN for regression to quantify NDs from spectra. We noted a large variation in ND in our samples, which may have inhibited the ability of a regressor CNN to quantify NDs from spectra.

Monitoring of cell density, the fraction of extracellular components such as collagens, and the degree of cellular infiltration is essential to diagnosis and treatment planning for certain types of heart disease. Our studies indicate that LSS can provide a rapid means of tissue characterization in the heart. There is no destructive process required for this tissue characterization. Due to the simplicity of the developed LSS probe, it could be single-use disposable. This constitutes an advantage over many existing modalities such as FCM or OCT, where probes are expensive each due to their technical complexity and require sterilization protocols.^38^

Potential applications of the developed LSS system include the diagnosis of heart diseases. Decreases in myocyte density and increases in cellular infiltrates are a hallmark of hypertrophy and myocarditis, respectively.^39^ Also, fibrosis and infarctions are characterized by changes in ND. Another application of the developed system could be surveillance of heart transplant patients for cellular rejection. The gold standard for surveilling heart transplant rejection is performing an endomyocardial biopsy.^40^ First developed in the 1960s and 1970s, the procedure requires removing small amounts of tissue from the ventricular septum. Endomyocardial biopsy is associated with significant complications ^41^^,^^42^ and is often performed ten to twenty times in the first year after a heart transplant. LSS has the potential to allow for cardiac transplant rejection surveillance without the need for endomyocardial biopsy and tissue removal.^43^

The results of our experiments with a reduced resolution spectral dataset indicate that a spectrometer with nanometer wavelength resolution is not necessary to achieve a high level of accuracy for ND prediction via CNN. The LSS system could be based on spectrometers with lower wavelength resolution. This finding can inform the cost-effective design of future LSS systems.

A limitation of our work is that we ignored intensity differences and normalized the spectra to the mean intensity before analyses. Our motivation was to reduce the effects of attenuation of spectra due to bending of the optical fibers and suboptimal connection of fibers to the spectrometer. Also, we applied a simple Gaussian filter to remove noise. The filter parameters were determined by visual inspection of a Fourier analysis of a representative sample of the dataset. The standard deviation of 20 spectral measures was selected due to it removing the majority of the noise from the signal while still preserving the more subtle fluctuations in the spectra. More advanced filters might improve the reduction of noise in the spectra. We note also limitations regarding the specific approach to machine learning used in this study. We only investigated CNNs for classification. Other methods of machine learning, such as fully connected neural networks and k-means clustering, could yield similar or higher levels of accuracy and performance. We also acknowledge the limitations associated with the size of the dataset used for this study. A larger dataset could improve the performance of the networks trained in this study, potentially resulting in a successful regression estimation of NDs from LSS data. Due to the size of the datasets used, our networks could overfit the training dataset during the training process. We used LOOCV, where the hyperparameter variation was performed on data that were separate from the training and testing datasets, to prevent biasing the network to the testing dataset. We also kept the number of trainable parameters small in our networks, <500, to mitigate overfitting during the training process. Although a minimal hyperparameter search was performed, we acknowledge that this study was focused on whether the LSS system combined with neural networks would be capable of characterizing tissues according to nuclear densities, not on producing a fully and thoroughly optimized network for performing this characterization. Future work will focus on improving and refining the LSS system, the associated data processing pipeline, and the machine learning techniques used to classify the data.

## 5 Conclusions

Two machine learning approaches, spectral clustering and CNNs, allowed us to identify, with high accuracy, a range of NDs in cardiac tissues using spectra gathered by a customized LSS system. The described system is of low technical complexity and cost. The combination of LSS and machine learning constitutes a useful and affordable tool for the diagnosis of cardiac tissues for diseases hallmarked by microarchitectural changes. The presented approach has applications in monitoring and diagnosing heart diseases such as hypertrophy, fibrosis, and myocarditis. A further application is transplant rejection surveillance in lieu of traditional endomyocardial biopsy.

## Code, Data, and Materials Availability

The code for processing and analyses in this study is available from the corresponding author upon reasonable request. The data produced in this study are available from the University of Utah Research Data Repository at the following link: https://doi.org/10.7278/S50D-FV1Q-BYNS
